# The role of cardiovascular magnetic resonance in women with suspected CAD: a CE-MARC substudy

**DOI:** 10.1186/1532-429X-14-S1-O94

**Published:** 2012-02-01

**Authors:** John P Greenwood, Manish Motwani, Neil Maredia, John Younger, Julia Brown, Jane Nixon, Colin Everett, Petra Bijsterveld, John P Ridgway, Aleksandra Radjenovic, Catherine J Dickinson, Stephen G Ball, Sven Plein

**Affiliations:** 1 Multidisciplinary Cardiovascular Research Centre (MCRC) & Leeds Institute of Genetics, Health and Therapeutics, University of Leeds, Leeds, UK; 2 Department of Cardiology, Royal Brisbane and Women's Hospital, Brisbane, QLD, Australia; 3 Clinical Trials Research Unit, University of Leeds, Leeds, UK; 4 Department of Medical Physics, Leeds General Infirmary, Leeds, UK; 5 Department of Nuclear Cardiology, Leeds General Infirmary, Leeds, UK

## Summary

The CE-MARC study is the largest, prospective evaluation of cardiovascular magnetic resonance (CMR) in patients with suspected coronary artery disease (CAD). This predefined CE-MARC substudy compared the diagnostic performance of CMR and single-photon emission computed tomography (SPECT) in the female cohort.

## Background

Coronary artery disease (CAD) is the leading cause of death in women but despite this they are often underrepresented in non-invasive imaging studies. Furthermore, the use of myocardial perfusion imaging in women presents challenges not encountered in men including a low premenopausal prevalence of CAD, more atypical symptoms, a different pattern of disease (more frequent single-vessel disease and intermediate grade stenosis), breast attenuation artefacts and smaller heart size. This substudy aimed to compare the diagnostic performance of CMR and single-photon emission computed tomography (SPECT) in the female cohort of the CE-MARC study [1].

## Methods

CE-MARC was a prospective study of 752 patients with suspected CAD. All patients were scheduled to undergo CMR and SPECT followed by invasive coronary angiography (the reference standard). CMR comprised adenosine stress/rest perfusion, cine imaging, late gadolinium enhancement and MR coronary angiography. Gated adenosine stress/rest SPECT was performed using 99mTc tetrofosmin. Visual analysis was performed on a per patient basis. For this pre-defined substudy, the diagnostic accuracy of CMR and SPECT to detect significant CAD in the female cohort (n = 281) was compared using McNemar's Chi-Squared Test and Leisenring’s Generalised Score Statistic. In a secondary analysis, receiver operating characteristic curves were generated for the stress perfusion CMR component and SPECT (using a summed stress scores for both).

## Results

**Figure 1:**
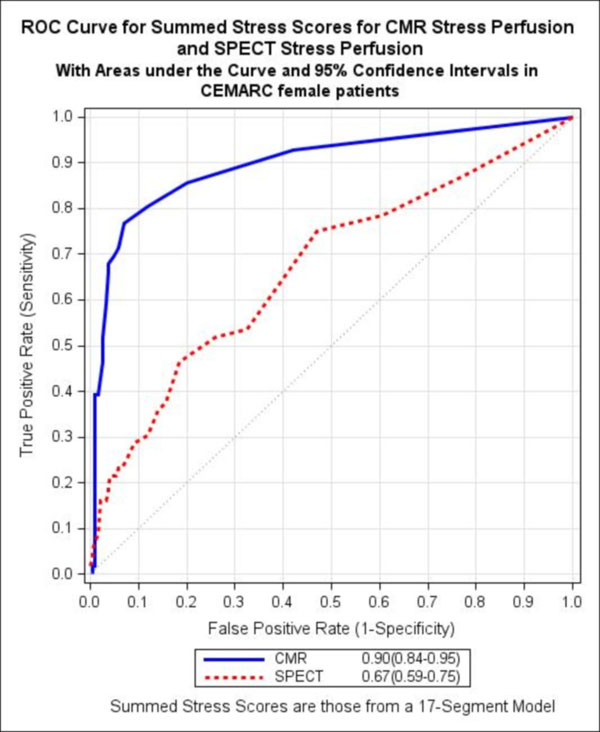
The AUC was significantly higher for stress perfusion CMR than for SPECT (AUC: 0.90 vs. 0.67; p<0.001)

235 female patients had interpretable CMR, SPECT and angiography. The prevalence of significant CAD was 22.6% (1VD 14.9%; 2VD 6.0%; 3VD 1.7%). The sensitivity of a multi-parametric CMR study was 88.7% (95%CI: 77.4-94.7), specificity 83.5% (95%CI: 77.4-88.2), positive predictive value (PPV) 61.0% (95%CI: 49.9-71.2) and negative predictive value (NPV) 96.2% (95%CI: 92.0-98.2). For SPECT the sensitivity was 50.9% (95%CI: 37.9-63.9), specificity 84.1% (95%CI: 78.1-88.7), PPV 48.2% (95%CI: 35.7-61.0) and NPV 85.5% (95%CI: 79.6-89.9). The differences between the sensitivity and NPV of CMR and SPECT were highly significant (χ2=18.18, 1df. P<0.001 and χ2=19.63, 1df. P<0.001 respectively); the difference between the PPVs was also significant (χ2=3.95, 1df. P=0.0468) but the specificities were not significantly different (χ2=0.02, 1df. P=1.000). In the secondary analysis, stress perfusion CMR (AUC: 0.90, 95%CI 0.84-0.95) significantly out-performed SPECT (AUC: 0.67, 95%CI 0.59-0.75) (P<0.001; Fig 1).

## Conclusions

CMR has significantly greater sensitivity, NPV and PPV compared to SPECT for the detection of CAD in women, but similar specificity. These findings in combination with an absence of ionising radiation exposure mean that CMR should be considered the preferred non-invasive imaging test for females with suspected CAD.<p>

## Funding

CE-MARC was funded by a British Heart Foundation Programme Grant (RG/05/004).
